# Quantification of Species-Preferential Micropylar Chemoattraction in *Arabidopsis* by Fluorescein Diacetate Staining of Pollen Tubes

**DOI:** 10.3390/ijms23052722

**Published:** 2022-02-28

**Authors:** Takuya T. Nagae, Hidenori Takeuchi, Tetsuya Higashiyama

**Affiliations:** 1 Division of Biological Science, Graduate School of Science, Nagoya University, Furo-cho, Chikusa-ku, Nagoya 464-8602, Japan; takuya.t.nagae@gmail.com; 2 Institute for Advanced Research, Nagoya University, Furo-cho, Chikusa-ku, Nagoya 464-8601, Japan; hidenori.takeuchi@itbm.nagoya-u.ac.jp; 3 Institute of Transformative Bio-Molecules (WPI-ITbM), Nagoya University, Furo-cho, Chikusa-ku, Nagoya 464-8601, Japan; 4 Department of Biological Sciences, Graduate School of Science, The University of Tokyo, Tokyo 113-0033, Japan

**Keywords:** species preferentiality, pollen tube attraction, fluorescein diacetate, Brassicaceae, *Arabidopsis lyrata*

## Abstract

Sexual reproduction between males and females of the same species is essential for species maintenance. Ovular micropylar guidance, the last step of pollen tube guidance in angiosperms, contributes to species-preferential reproduction. Previous studies using semi-in vivo attraction assays showed that species-preferential attractant peptides are secreted from the ovule through its micropyle. However, conventional semi-in vivo assays usually depend on transgenic pollen tubes expressing a fluorescent protein to determine whether the tubes are attracted to the ovule to precisely penetrate the micropyle. Here, we found that fluorescein diacetate (FDA) staining was suitable for evaluating the micropylar guidance rate of non-transgenic pollen tubes in semi-in vivo conditions. Micropylar guidance was quantified for ovules and pollen tubes of *Arabidopsis thaliana* and *Arabidopsis lyrata* by combining FDA staining with modified semi-in vivo assays. Our results using the simple staining method showed that the ovules of each species secrete species-preferential attractants, and that pollen tubes respond more strongly to attractants of their own species compared with those of closely related species. LURE-type CRP810 attractant peptides were shown to be responsible for micropylar attraction of *A. thaliana* in the semi-in vivo assay. The POLLEN-SPECIFIC RECEPTOR-LIKE KINASE 6 (PRK6) receptor for LURE1, as well as an unidentified receptor for other LURE-type attractants, are involved in the species-preferential response of these two *Arabidopsis* species.

## 1. Introduction

Sexual reproduction contributes to genetic diversity but accidental hybridization of species is possible. Genome-encoded prezygotic reproductive barriers have been reported, which prevent cross-species fertilization and increase the efficiency of conspecific fertilization in the presence of other species [1]. During the evolution of plants, the haploid generation (the gametophyte) has been progressively reduced to the point that in flowering plants, the gametophytes, embryo sac, and pollen (the female and male gametophytes, respectively) consist of just a few cells. The embryo sac is embedded in the ovule, which is further enclosed by the ovary of the pistil. Following pollination on the stigma (a receptive tissue of the pistil), the pollen extrudes a tubular cell called the pollen tube, delivering sperm cells to the embryo sac. Pollination carries a risk of crossing with other species, but complex pollen–pistil interactions enable specific or preferential acceptance of sperm cells of the same species. Molecules involved in species recognition have been reported in the context of pollination and pollen tube growth, pollen tube guidance, and pollen tube rupture for sperm delivery [2,3,4,5,6]. Pollen grains and tubes of distantly related species tend to stop germination and growth at earlier stages, while those of closely related species proceed to later stages [7,8,9]. Even in pollination of closely related species, the efficiency of sperm delivery tends to decrease, especially when there is competition with pollen tubes of the conspecific species [5].

Pollen tube attraction to the ovule is a major step in species recognition that can preferentially guide pollen tubes of the same species in the presence of pollen tubes of closely related species. The synergid cell on the side of the egg cell is the source of the ovular attraction signal that guides the pollen tube to its final destination, i.e., the embryo sac [10]. This last step of pollen tube guidance is called micropylar attraction. Attractant peptides specifically or predominantly expressed in the synergid cell have been reported. In eudicots, such as *Torenia fournieri* [11] and *Arabidopsis thaliana* [12], defensin-like pollen tube attractant peptides, referred to as LURE-type, have been identified. In monocots, such as *Zea mays*, other types of attractant peptides, referred to as EA1-type, have been identified [13]. Following introduction of the *A. thaliana LURE1.2* (*AtLURE1.2*) gene into *Torenia* [12] and the maize *EA1* gene into *Arabidopsis* [14], the transformed ovules were shown to attract pollen tubes of *Arabidopsis* and maize, respectively. Alteration of species recognition was also achieved by introducing the receptor gene of *Arabidopsis* LURE1, i.e., *Arabidopsis POLLEN-SPECIFIC RECEPTOR-LIKE KINASE* (*PRK6*) and *MALE DISCOVERER1* (*MDIS1*), into *Capsella rubella* [15,16].

Species preference for signaling via LURE-type attractants has been studied in *Torenia* and *Arabidopsis* [12,17]. CRP1 of *Torenia concolor* (TcCRP1), a homolog of *Torenia fournieri* LURE1 (TfLURE1), was shown to be specifically expressed in synergid cells, and to attract cultured pollen tubes [17]. Eight amino acid substitutions among the entire 70 amino acid sequence were identified. Recombinant TfLURE1 and TcCRP1 showed preferential attraction for *T. fournieri* and *T. concolor* pollen tubes, respectively [17], consistent with the preferential attraction for cultured ovules and pollen tubes of these species [18]. *LURE1* genes of *A. thaliana* and *A. lyrata* form species-specific clusters (*AtLURE1.1–1.6* and *AlLURE1.1–1.10* genes, respectively) [12]. Recombinant AtLURE1.2 was shown to preferentially attract pollen tubes of *A. thaliana* compared with *A. lyrata* pollen tubes, in conditions where *A. lyrata* pollen tubes are sufficiently attracted by *A. lyrata* LURE1 peptides. At least 19 amino acid substitutions were identified in AtLURE1.2 peptides, compared with AlLURE1 peptides [12].

In the *A. thaliana* Columbia strain, 10 *CYSTEINE-RICH PEPTIDE 810* (*CRP810*) genes, *CRP810_1.1*–*1.4*/*LURE1.1*–*1.4*, *CRP810_2.2*/*TICKET2*, *CRP810_2.3*/*XIUQIU4*/*TICKET3*, *CRP810_3.1*–*3.2*/*LURE1.7*–*1.8*, and *CRP810_4*–*5*/*XIUQIU1*–*2*, have been reported to encode functional LURE-type defensin-like attractant peptides [4,5,12]. These genes are specifically expressed in the synergid cell under the control of the synergid cell-specific transcription factor MYB98 [4,5,12,19,20,21]. *LURE1s* with a species-specific gene cluster are thought to be involved in conspecific pollen tube guidance for the combination of *A. thaliana* and *A. lyrata* [12]. CRP810_2.2/TICKET2 (TIC2) have recently been identified as species-specific attractants [4]. *A. thaliana* TIC2 and the orthologous TIC of *A. lyrata* and *C. rubella* can attract their own species pollen tubes, but not those of closely related species [4]. In short, multiple attractants contribute to pollen tube attraction in a species-preferential manner.

However, a few issues remain to be addressed for a complete understanding of species-preferentiality in ovular attraction. First, the contribution of multiple attractant peptides secreted from the synergid cell and receptors of the pollen tube to species preferentiality is unclear [22]. While the PRK6 receptor, which is localized to the plasma membrane at the pollen tube tip, is a major receptor for LURE1 peptides, the existence of additional receptors for XIUQIU and TICKET peptides has been suggested [5,23]. For a better understanding of species-preferential pollen tube attraction in *Arabidopsis*, it will be important to quantitatively determine how LURE-type CRP810 peptides and their receptors are involved in species-preferential attraction. Second, further quantification of preferential attraction by the ovule is needed. The embryo sac is embedded in the ovule, except in unique plant species such as *Torenia*, which has a protruding embryo sac. The labeling of pollen tubes is necessary to quantify ovule-targeting frequency. In non-model species such as *A. lyrata*, however, preparation of a marker line, i.e., a transformant with a fluorescent protein gene, for visualization of the paths of pollen tubes around the ovule is difficult. To precisely assess the targeting of ovules by *A. lyrata* pollen tubes, the dragging method has been used, in which targeting was confirmed by micromanipulation of ovules [24]. A conventional chemical staining method would be useful to study species preferentiality and specificity in ovular attraction of non-model species like *A. lyrata*.

In the present study, we developed a novel chemical staining method to visualize ovule targeting by the pollen tube. Fluorescein diacetate (FDA) staining prior to observation could readily visualize ovule targeting; this method was combined with cultivation methods to detect species differences in the micropylar attraction signal. Moreover, CRP810 peptides were shown to be responsible for the attraction by cultured ovules (semi-in vivo assay). Our results suggest that PRK6, as well as an unknown receptor for other CRP810 peptides, was involved in species-preferential pollen tube attraction.

## 2. Results

### 2.1. FDA Staining Enables to Directly Observe Pollen Tube Attracted into Ovules

**Figure 1:**
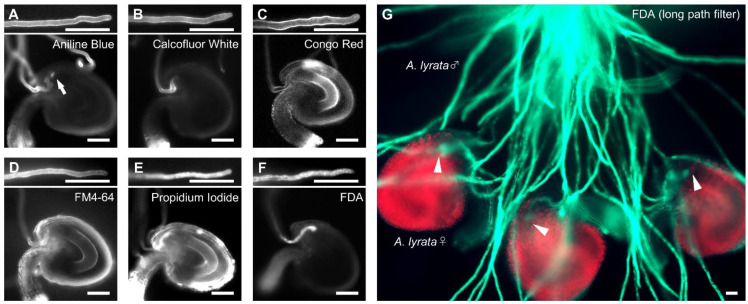
Chemical stains for pollen tubes and ovules of *Arabidopsis* species on medium. (**A**–**F**) The upper parts of each figure show representative images of a pollen tube stained with 0.1% aniline blue (**A**), 0.001% calcofluor white (**B**), 0.4% Congo red (**C**), 5 µM FM4-64 (**D**), 50 µM propidium iodide (PI) (**E**), and 5 µM fluorescein diacetate (FDA) (**F**). The lower parts of each figure show representative images of a stained pollen tube attracted into the ovule. An arrow in (**A**) indicates the filiform apparatus stained with aniline blue. (**G**) Semi-in vivo assay of *A. lyrata* pollen tubes and ovules. Pollen tubes were stained with 5 µM FDA. Arrowheads in (**G**) indicate the tips of the attracted pollen tubes. Each image was acquired after staining for 5 min. Scale bars, 20 µm.

To develop a chemical staining method to visualize pollen tubes attracted to the ovule, to reach the embryo sac [25], we tested six fluorescent dyes previously used for pollen tube staining in vitro and in vivo: aniline blue, calcofluor white, Congo red, propidium iodide (PI), FM4-64, and FDA [26,27,28,29,30,31]. First, we stained *A. thaliana* pollen tubes growing on the medium with each dye for five minutes under non-fixed conditions. After applying each dye, pollen tubes stopped growing; however, they were successfully stained by all dyes tested (Figure 1A–F). Aniline blue, calcofluor white, and Congo red stained the cell wall of pollen tubes, as expected; these dyes specifically bind callose (β-1,3-glucan), cellulose, and carbohydrates, respectively (Figure 1A–C) [32,33]. FM4-64 stained the plasma membrane of pollen tubes (Figure 1D). However, the tips of pollen tubes attracted into ovules were not confirmed by these dyes (Figure 1A–D). Aniline blue also stained the filiform apparatus enriched in callose (Figure 1A). PI and FDA stained the cytoplasm of pollen tubes (Figure 1E,F). Pollen tube tips in the micropyle of ovules were observed by PI and FDA staining, although PI-stained ovules stained more non-specifically than with FDA (Figure 1E,F). We also confirmed that FDA staining allowed visualization of *A. lyrata* pollen tubes in the micropyle of *A. lyrata* ovules (Figure 1G). These results indicate that FDA staining is suitable for direct quantification of pollen tube attraction.

### 2.2. Arabidopsis Ovules Attract Pollen Tubes in a Species-Preferential Manner

**Figure 2:**
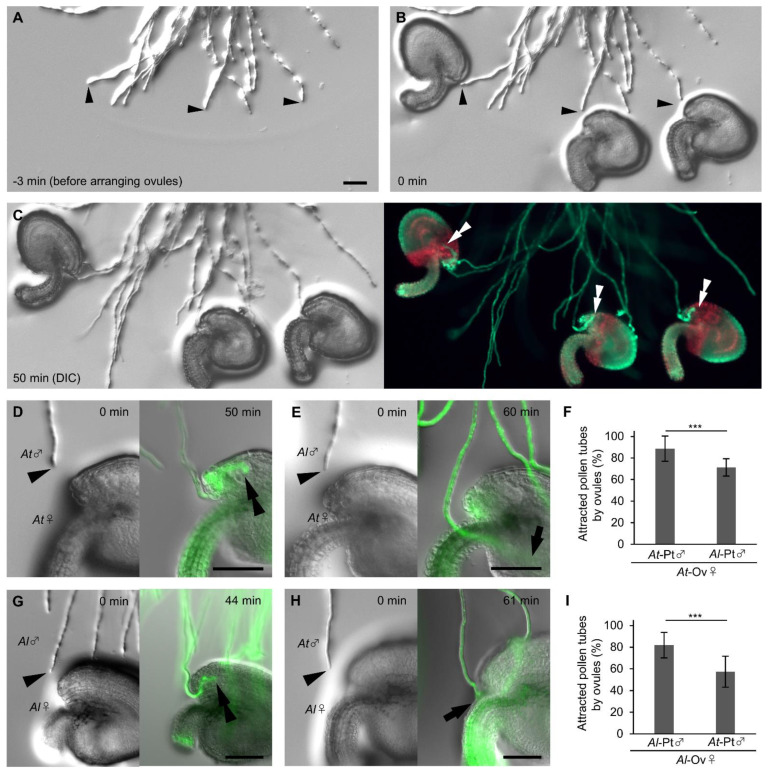
Precise ovule arrangement assay for *Arabidopsis* species. (**A**–**C**) Procedure for the ovule arrangement assay. Each differential interference contrast (DIC) image shows pollen tubes before arranging ovules (**A**), pollen tubes after arranging ovules immediately (**B**), and pollen tubes attracted into ovules (**C**, **left**). Pollen tubes were stained with 5 µM FDA for 5 min (**C**, **right**). (**D**–**F**) Ovule arrangement assay with *A. thaliana* (*At*) and *A. lyrata* (*Al*) pollen tubes for *A. thaliana* ovules. Rates of pollen tube (Pt) attraction of *A. thaliana* (**D**) and *A. lyrata* (**E**) by *A. thaliana* ovules (Ov) are shown in (**F**). (**G**–**I**) Ovule arrangement assay with *A. lyrata* and *A. thaliana* pollen tubes for *A. lyrata* ovules. Rates of pollen tube attraction of *A. lyrata* (**G**) and *A. thaliana* (**H**) are shown in (**I**). Arrowheads indicate the tips of pollen tubes immediately after arranging the ovules. Double arrowheads indicate the tips of attracted pollen tubes. Arrows indicate the tips of unattracted pollen tubes. Each assay was replicated five times with 11–16 ovules. Fisher’s exact test, *** p < 0.001. Data are mean ± standard deviation (SD). Scale bars, 50 µm.

To elucidate whether ovules secrete signals that preferentially attract pollen tubes, we quantified and compared pollen tube attraction rates between *A. thaliana* and *A. lyrata* ovules. In the conventional semi-in vivo assay [25], in which ovules and pollinated cut styles are arranged at the onset of cultivation, the number of pollen tubes approaching each ovule and the approaching angle of each pollen tube are unable to control, resulting in variable results. We therefore developed a modification which we termed the precise ovule arrangement assay (Figure 2A–C; Supplementary Figure S1). In the precise ovule arrangement assay, at 4.5 h after pollination on pistils and then cultivation of cut styles, we arranged each mature ovule diagonally in front of each pollen tube for examination. To prevent pollen tubes from accidentally entering into the micropyle, we adjusted the distance between the tip of a pollen tube and the micropyle of an ovule, and the orientation of each ovule (Supplementary Figure S1); the micropyle was settled at a distance of 50 to 100 µm from the pollen tube tip with an angle of 20 to 40 degrees, with the orientation of the ovule so that the pollen tube and the funiculus become paralleled. We arranged 3–6 ovules for each cut style. We performed FDA staining 1 h after arranging the ovules and quantified the pollen tube attraction rate (Figure 2D–I). The pollen tubes that changed direction toward the ovules but did not enter into the ovules, as shown in Figure 2E, were classified as unattracted. *A. thaliana* ovules attracted more *A. thaliana* pollen tubes (88.7 ± 11.7%, *n* = 135) than *A. lyrata* pollen tubes (70.5 ± 8.1%, *n* = 112; Fisher’s exact test, *** *p* < 0.001; Figure 2D–F). *A. lyrata* ovules attracted more *A. lyrata* pollen tubes (81.9 ± 12.8%, *n* = 84) than *A. thaliana* pollen tubes (57.4 ± 15.5%, *n* = 96; Fisher’s exact test, *** *p* < 0.001; Figure 2G–I). A similar tendency was confirmed with the conventional semi-in vivo assay, although the difference was less clear (Supplementary Figure S2). *A. thaliana* ovules attracted more *A. thaliana* pollen tubes (90.0 ± 7.0%, *n* = 60) than *A. lyrata* pollen tubes (77.8 ± 12.6%, *n* = 76; Fisher’s exact test, *p* = 0.067; Supplementary Figure S2A–C); *A. lyrata* ovules attracted more *A. lyrata* pollen tubes (71.6 ± 13.9%, *n* = 61) than *A. thaliana* pollen tubes (53.3 ± 16.2%, *n* = 60; Fisher’s exact test, *p* = 0.039; Supplementary Figure S2D–F). These results indicate that the ovules of *A. thaliana* and *A. lyrata* secrete signals to attract pollen tubes in a species-preferential manner.

### 2.3. Semi In Vivo Pollen Tube Attraction Is Regulated by PRK6 and CRP810_1, CRP810_2, CRP810_3, and CRP810_4 Proteins

**Figure 3:**
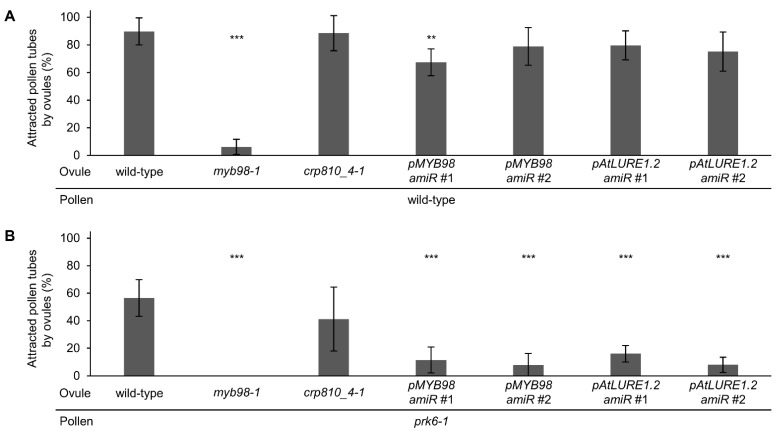
Precise ovule arrangement assay for pollen tubes of wild-type and *prk6-1* with ovules of wild-type, *myb98*, *crp810_4-1*, and *CRP810* genes knockdown lines. (**A**) Attraction rates of wild-type pollen tubes for ovules of wild-type, *myb98-1*, *crp810_4-1*, and *crp810_4-1* which transformed *CRP810_1-3* amiRNA vectors (pPZP211-pMYB98::amiR-CRP810_1s-CRP810_2s-CRP810_3s and pPZP211-pAtLURE1.2::amiR-CRP810_1s-CRP810_2s-CRP810_3s). The strains of *crp810_4-1* transformed *CRP810_1-3* amiRNA vectors were labeled as *pMYB98amiR*#1, #2 and *pAtLURE1.2amiR*#1, #2 (independent lines). (**B**) Attraction rates of *prk6-1* pollen tubes for ovules of wild-type, *myb98-1*, *crp810_4-1*, and *crp810_4-1* transformed *CRP810_1-3* knock down vector. Each assay was replicated eight times with 10–17 ovules. Dunnett’s two-sided test compared with wild-type ovule, ** p < 0.01, *** p < 0.001. Data are mean ± SD.

Most of the recombinant LURE-type CRP810 proteins showed pollen tube attraction activity [22]; however, it was unclear whether semi-in vivo pollen tube attraction was controlled by CRP810 family proteins or other secreted molecules. To assess whether semi-in vivo pollen tube attraction is mainly regulated by CRP810 family proteins, we designed two amiRNA vectors which simultaneously target six CRP810_1 genes, three CRP810_2 genes, and two CRP810_3 genes (Supplementary Figure S3). We introduced the vectors into the *crp810_4-1*/*xiuqiu1* mutant so that predominantly expressed CRP810_1 to 4 are downregulated. We performed the precise ovule arrangement assay using the knockdown lines. First, as the control, we quantified pollen tube attraction for *myb98* ovules. Wild-type ovules frequently attracted wild-type pollen tubes (89.7 ± 9.8%, *n* = 98), while *myb98-1* ovules rarely attracted wild-type pollen tubes (6.2 ± 5.4%, *n* = 108; Dunnett’s test compared with wild-type, *p* < 0.001; Figure 3A), consistent with a previous report [34]. Next, *crp810_4-1*/*xiuqiu1* mutant ovules with or without amiRNAs targeting CRP810_1s, CRP810_2s, and CRP810_3s were examined. *crp810_4-1*/*xiuqiu1* mutant ovules attracted wild-type pollen tubes, as well as wild-type ovules (*crp810_4-1*, 88.5 ± 12.7%, *n* = 99). However, the knockdown lines with amiRNA attracted slightly fewer pollen tubes (*pMYB98-amiR*#1, 67.3 ± 9.8%, *n* = 96; *pMYB98-amiR*#2, 78.9 ± 13.6%, *n* = 93; *pAtLURE1.2-amiR*#1, 79.6 ± 10.5%, *n* = 91; *pAtLURE1.2-amiR#2*, 75.1 ± 14.22%, *n* = 95; Figure 3A). To shutoff signaling via the PRK6 receptor, we repeated the analysis using *prk6-1* pollen tubes, which cannot respond to CRP810_1s/LURE1s on the medium. Wild-type and *crp810_4-1*/*xiuqiu1* ovules attracted about half of the *prk6-1* pollen tubes (wild-type, 56.5 ± 14.2%, *n* = 95; *crp810_4-1*/*xiuqiu1*, 41.2 ± 25.0%, *n* = 96; Dunnett’s test compared with wild-type, *p* = 0.078, Figure 3B). By contrast, ovules of the knockdown lines rarely attracted *prk6-1* pollen tubes (*pMYB98-amiR*#1, 11.6 ± 9.4%, *n* = 99; *pMYB98-amiR*#2, 7.8 ± 8.4%, *n* = 100; *pAtLURE1.2-amiR*#1, 16.1 ± 5.9%, *n* = 109; *pAtLURE1.2-amiR*#2, 8.1 ± 5.5%, *n* = 93; Dunnett’s test compared with wild-type, *** *p* < 0.001, Figure 3B). No attraction was observed with the combination of *myb98-1* ovules and *prk6-1* pollen tubes (0%, *n* = 100; Figure 3B). These results suggest that CRP810 family members and PRK6 are the main contributors to pollen tube attraction, and these proteins may be responsible for species-preferential pollen tube attraction.

### 2.4. The Ovule Competition Assay Is More Sensitive for Detecting Species Differences in the Attraction Signal

**Figure 4:**
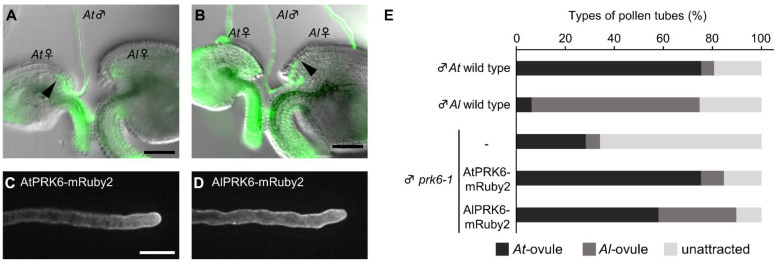
Ovule-competition assay for *Arabidopsis* species. (**A**,**B**) Ovule competition assay for a pollen tube of *A. thaliana* (**A**) and *A. lyrata* (**B**) with *A. thaliana* and *A. lyrata* ovules. Pollen tubes were stained with FDA. Arrowheads indicate the tips of attracted pollen tubes. Scale bars, 50 µm. (**C**,**D**) Representative single-plane confocal images of *A. thaliana* pollen tubes expressing AtPRK6-mRuby2 (**C**) and AlPRK6-mRuby2 (**D**). Scale bars, 20 µm. (**E**) Rates of attracted pollen tubes revealed by ovule competition assay with *A. thaliana* and *A. lyrata* ovules. Black bars show attraction to *A. thaliana* ovules; dark gray bars show attraction to *A. lyrata* ovules. Light gray bars show pollen tubes not attracted to either ovule. Each assay was replicated at least five times with 10–18 pairs of ovules.

While we showed using the ovule arrangement assay that *Arabidopsis* ovules attracted more pollen tubes of the same species (Figure 2; Supplementary Figure S1), it was difficult to quantitatively analyze the factors contributing to the species preferentiality. We thus employed an ovule competition assay [12] with *A. thaliana* ovules (*At*-ovules) and *A. lyrata* ovules (*Al*-ovules). At 4.5 h after the start of cultivation, we arranged a pair of *A. thaliana* and *A. lyrata* ovules opposite each other in front of a pollen tube, with a 50 to 100 µm gap between their micropyles (Figure 4A). The ovules were set at an angle of about 30 degrees with a distance of 50 to 100 µm from the pollen tube tip to the micropyle. We expected this method to allow us to detect species preferentiality more accurately, because it can balance the effect of non-species-preferential attractants secreted from ovules. We analyzed whether pollen tubes were more attracted to their own ovules than the related ovules using this assay. *A. thaliana* pollen tubes were more attracted by *A. thaliana* ovules than *A. lyrata* ovules (*At*-ovules, 75.4 ± 19.6%; *Al*-ovules, 5.2 ± 7.3%; unattracted, 19.3 ± 13.4%; number of ovule pairs = 57, Figure 4A,E). *A. lyrata* pollen tubes were more attracted by *A. lyrata* ovules than *A. thaliana* ovules (*At*-ovules, 63.4 ± 4.3%; *Al*-ovules, 68.4 ± 6.1%; unattracted, 25.3 ± 6.5%; number of ovule pairs = 79, Figure 4B,E). These data suggest that each ovule secretes species-preferential attractants, and that pollen tubes recognize these attractants to regulate their direction of growth.

### 2.5. PRK6 and an Unidentified Receptor(s) of A. thaliana and A. lyrata Recognize Attractants of Their Own Species

To investigate whether PRK6 of both *A. thaliana* and *A. lyrata* contributes to species-preferential attraction of pollen tubes, we generated a *prk6-1* expressing PRK6 orthologue of *A. lyrata* (AlPRK6) as an mRuby-2 fusion under the control of its own promoter. AlPRK6-mRuby2 was localized on the plasma membrane of pollen tubes, similar to the localization of AtPRK6-mRuby2 shown previously (Figure 4C,D) [15]. In the ovule competition assay, pollen tubes of *prk6-1* expressing AtPRK6-mRuby2 were more attracted by *A. thaliana* ovules than those of *A. lyrata*, similar to wild-type *A. thaliana* pollen tubes (*At*-ovules, 75.3 ± 9.2%; *Al*-ovules, 9.4 ± 10.0%; unattracted, 15.3 ± 7.8%; number of ovule pairs = 85; Figure 4E). By contrast, pollen tubes of *prk6-1* expressing AlPRK6-mRuby2 were less attracted to *A. thaliana* ovules and more attracted to *A. lyrata* ovules (*At*-ovules, 58.0 ± 20.3%; *Al*-ovules, 31.8 ± 18.6%; not attracted, 10.2 ± 11.0%; number of ovule pairs = 88; Figure 4E). Attraction rate of *prk6-1* pollen tubes expressing AlPRK6-mRuby2 to for *A. lyrata* ovules was statistically significantly increased compared to pollen tubes expressing AtPRK6-mRuby2 (Fisher’s exact test, *p* < 0.001). These results indicate that PRK6 of both species is a key receptor recognizing species-preferential attractants. It should be noted that pollen tubes of *prk6-1* expressing AlPRK6-mRuby2 were more attracted to *A. thaliana* ovules (Figure 4E). This was consistent with the species-preferential attraction of *prk6-1* pollen tubes, although the overall pollen tube attraction rate decreased (*At*-ovules, 28.4 ± 13.3%; *Al*-ovules, 5.7 ± 6.7%; not attracted, 66.0 ± 13.0%; number of ovule pairs = 88; Figure 4E). This suggests that pollen tubes possess PRK6-independent pathway(s) to recognize species-preferential attractants.

## 3. Discussion

Here, we showed that FDA staining is a powerful tool to visualize pollen tube cell growth semi-in vivo, which will be useful in the study of species-preferentiality/specificity in ovule-targeting even in non-model species. FDA was developed as a fluorogenic substrate to visualize living cells [35]. Enzymatic activity and cell membrane integrity of living cells are required to activate fluorescence in the cell. FDA preferentially stained pollen tube cells were compared with ovular cells in semi in vivo condition, which enabled us to clearly trace the pollen tube tip even in the ovule. FDA was not sufficient to preferentially stain pollen tubes growing in vivo due to staining of female cells (data not shown). However, FDA has a relatively simple xanthene structure, which can be modified by chemical synthesis. It would be interesting to modify the structure of FDA to increase specificity for the pollen tube in the future.

By combining FDA staining and the precise ovule arrangement assay, we could quantitatively demonstrate species differences in micropylar attraction of *A. thaliana* and *A. lyrata* ovules (Figure 2). Reciprocal analysis showed that the ovules of each species attracted more pollen tubes of the same species, supporting the notion that pollen tubes of both species were competent in terms of attraction. These results suggest that the difference in micropylar attraction signal was successfully detected semi-in vivo in these closely related species.

Semi-in vivo micropylar attraction was shown to depend on CRP810 attractant peptides and PRK6 receptor signaling pathways (Figure 3). Knockout/knockdown of these genes largely abolished the semi-in vivo pollen tube attraction. This is not likely to be the case for pollen tube guidance in vivo; pollen tube guidance was still observed in vivo under the same knockout/knockdown conditions (data not shown), consistent with the report that knockout of all functional LUREs and XIUQIUs impaired pollen tube guidance only slightly in vivo [5]. The species difference detected semi-in vivo appears to depend on differences in CRP810 peptides and the PRK6 receptor. Liu et al. (2021) used a dragging assay under semi-in vivo conditions to show that *lure1* null, but not *xiuqiu* null *A. thaliana* ovules exhibited decreased species-preferential attraction for pollen tubes of *A. thaliana* compared with those of *A. lyrata* [24]. This species preferentiality of *A. thaliana* was shown to depend on the LURE1/PRK6 pathway [24]. Consistent with this, our results showed that PRK6 of both *A. thaliana* and *A. lyrata* was involved in species-preferential micropylar attraction. PRK6 of *A. lyrata* was shown to complement LURE1 sensitivity and modify species preferentiality. A cocrystal structure of AtLURE1.2 and AtPRK6 showed that the amino acids critical for binding these proteins (Arg83 of AtLURE1.2 and Asp234 of AtPRK6) are conserved in *A. thaliana* and *A. lyrata* [23]. Other amino acids at their interface are suggested to contribute to the species preferentiality. Modeling analysis combined with mutational analysis of amino acids of LURE1/PRK6, performed using our sensitive assays, could clarify how species preferentiality arises from differences in LURE1/PRK6 in these species. Attraction by AtLURE1.7 and -1.8 (CRP810_3) was shown to be dependent on AtPRK6 [5]; therefore, it would also be of interest to examine how this class of LURE1 interacts with PRK6.

Species-preferential attraction by ovules was still observed in pollen tubes of *A. thaliana* in which PRK6 was swapped between species. This suggests that other CRP810 attractants, such as TIC2 [4] and its unidentified receptor, are involved in species-preferential micropylar attraction. MDIS receptor-like kinase, previously reported as a receptor for LURE1 [16], might be a candidate. Identification of the receptor(s) for other LURE-type CRP810 attractants will help elucidate how the species preferentiality of micropylar attraction of *Arabidopsis* species is controlled via the LURE1/PRK6 pathway. The methods developed in this study will allow for further detailed, quantitative analyses of species-preferential pollen tube attraction.

## 4. Materials and Methods

### 4.1. Plant Materials

*Arabidopsis thaliana* Columbia-0 (Col-0) was used as the wild-type plant. T-DNA insertion lines of *prk6-1* (SALK_129244) [15], *myb98-1* (SALK_020263) [36], *crp810_4-1* (SALK_105771), and *ms1* (SALK_055721) were obtained from Arabidopsis Biological Resource Center (ABRC) or Nottingham Arabidopsis Stock Centre (NASC). All T-DNA insertions were confirmed by genomic PCR (Supplementary Table S1). pAtPRK6::AtPRK6-mRuby2 in *prk6-1* lines were established previously [15]. *Arabidopsis lyrata* MN47 (accession CS22696) seed was originally provided by Akira Kawabe (Kyoto Sangyo University) [12].

### 4.2. Growth Conditions

Seeds were sterilized with 70% ethanol and sown on 0.8% (w/v) agar plates containing 2.3 g L^−1^ Murashige and Skoog salt (Duchefa Biochemie, Haarlem, The Netherlands), 1% sucrose, and 0.05% MES (pH 5.8). Plates with *A. thaliana* seeds were placed at 4 °C for 2 days and transferred to a growth chamber at 22 °C under 24 h light. Plates with *A. lyrata* seeds were placed at 4 °C for 2 weeks and transferred into a growth chamber at 22 °C under continuous light. Ten-day-old seedlings of *A. thaliana* and *A. lyrata* were transferred to soil and grown at 22 °C under continuous light. For vernalization, 6-week-old *A. lyrata* plants were incubated at 4 °C under 16 h light/8 h dark cycles for 3 weeks.

### 4.3. Pollen Tube-Ovule Interaction Assay

Pistils of the male sterile mutant *ms1* (Col-0 genetic background) were used as acceptors of *A. thaliana* and *A. lyrata* pollen grains. Pollen tubes were cultured on the medium through a cut style, as previously described [12] with some modifications. Briefly, the pollen germination medium (10% sucrose, 0.01% boric acid, 5 mM CaCl_2_, 1 mM MgSO_4_, 1.5% NuSieve™ GTG™ agarose (LONZA, Switzerland) and adjusted to pH 7.5 with KOH) [37] was poured into a mold made with 1-mm-thick silicone rubber and cover glasses. Pistils were pollinated under a stereomicroscope and cut at the bottom of styles with a 27-gauge needle (Terumo, Japan), and then placed horizontally on medium. For a conventional semi-in vivo assay, ovules were arranged with minutien pins (26002-20; Fine Science Tools, North Vancouver, BC, Canada) by hand under a stereomicroscope (SZX16; Olympus, Japan) and moist conditions, 10 min after placing the pollinated style. Three or four ovules per cut style were used to assess the interactions of pollen tubes. At 5.5 h after pollination at 22 °C in the dark, the entry of pollen tubes into micropyles was evaluated by silicon oil-based FDA staining of all placed ovules. For a precise ovule arrangement assay, at 4.5 h after pollination at 22 °C in the dark, ovules were placed diagonally in front of each pollen tube under a stereomicroscope in moist conditions. Ovules were settled at an angle of 20 to 40 degrees with a distance of 50 to 100 µm from the pollen tube tip to the micropyle. Three or six ovules per cut style were settled. At 1 h after placing the ovules, i.e., 5.5 h after pollination, the entry of pollen tubes into micropyles was evaluated for all placed ovules. For the ovule-competition assay, 4.5 h after pollination at 22 °C in the dark, *A. thaliana* and *A. lyrata* ovules were arranged opposite each other in front of a pollen tube, with a 50 to 100 µm gap between their micropyles, under a stereomicroscope in moist conditions. The ovules were settled at an angle of 20 to 40 degrees with a distance of 50 to 100 µm from the pollen tube tip to each micropyle. At 1 h after arranging the ovules, the entry of pollen tubes into micropyles was evaluated by silicon oil-based FDA staining of all placed ovules.

### 4.4. Chemical Florescence Staining of Pollen Tubes

For evaluation of fluorescent staining of pollen tubes on the medium and in the micropyle, aniline blue (415049; Merck, Germany), fluorescent brightener 28 (used as calcofluor white; F3543; Merck, Germany), Congo red (032-03922; Wako, Japan), PI (P4864; Merck, Germany), FM4-64 (F34653; Thermo Fisher Scientific, Waltham, MA, USA), and FDA (F7378; Merck, Germany) were prepared in the pollen germination medium without agarose. The final concentration of each dye is shown in Figure 1. At 5.5 h after pollination, 20 µL of each dye was dropped on the medium. After 5 min, staining of pollen tubes was observed with an Axio Imager A2 upright microscope (Zeiss, Jena, Germany) equipped with a cooled charge-coupled device (CCD) camera (Axiocam 506 color; Zeiss, Germany). Filter set 47 HE was used for aniline blue and calcofluor white. Filter set 31 was used for Congo red, PI, and FM4-64. Filter sets 09 and 38 were used for FDA. To quantify pollen tube attraction into ovules, pollen tubes were stained with 5 µM FDA dissolved in hydrated silicone oil (KF-96–100CS; Shin-Etsu Chemical, Japan). After 5 min of staining, pollen tubes were observed.

### 4.5. Vector Construction and Plant Transformation

For expression of the full-length AtPRK6 orthologue of *A. lyrata* (AlPRK6) as an mRuby2-fusion protein under the control of its own promoter, the genomic sequence of *AlPRK6* containing the promoter and coding region was amplified (primers: 5′-aggagtcgacGTTTTCAAGTTACAGAGAAAG-3′ and 5′-ttggcgcgccAAACTTTTACTTGTTCTATC-3′) from the genomic DNA of *A. lyrata*, and cloned into pPZP221Ru [15] using *Sal*I and *Asc*I sites. The construct was transformed into *prk6-1* plants using the floral dip method [37]. Transformed plants were selected on medium containing 100 mg mL^−1^ gentamicin.

To generate knockdown lines of *CRP810* genes, amiRNA vectors, which simultaneously target six *CRP810_1* genes, three *CRP810_2* genes, and two *CRP810_3* genes in one expression vector, were designed using the original system according to the established protocol (http://wmd.weigelworld.org, accessed on 18 January 2022) [38]. Binary vectors modified from pPZP211 by introducing the NOS terminator and *MYB98* (−1609 to −1) or *AtLURE1.2/CRP810_1.2* (−665 to −1) promoter were used for specific expression of amiRNA precursors in the synergid cell. Three amiRNAs (amiR-CRP810_1s, amiR-CRP810_2s, and amiR-CRP810_3s) were designed as shown in Supplementary Figure S3. Each amiRNA precursor cassette was generated by PCR using pRS300 (#22846; Addgene) as a template. Using a forward primer (5′-tcgcagatctCAAACACACGCTCGGAC-3′), a *Bgl*II site was added to the 5′ end of the PCR products. The PCR products of the amiRNA precursor sequences, amiR-CRP810_1s, amiR-CRP810_2s, and amiR-CRP810_3s, were digested with *Bgl*II and *Bam*HI (endogenous sites downstream of the amiRNA precursor sequence in pRS300) and sequentially and tandemly ligated into each pPZP211-based binary vector using the *Bam*HI site between the promoter and NOS terminator. The two resultant amiRNA vectors, pPZP211-pMYB98::amiR-CRP810_1s-CRP810_2s-CRP810_3s (HTv330, pMYB98amiR-CRP810_1-3) and pPZP211-pAtLURE1.2::amiR-CRP810_1s-CRP810_2s-CRP810_3s (HTv331, pAtLURE1amiR-CRP810_1-3), were transformed into *crp810_4-1* plants. Transformed plants were selected on medium containing 50 mg mL^−1^ kanamycin. For each line, T3 siblings obtained from T2 homozygous lines, which were confirmed based on kanamycin resistance, were used for the precise ovule arrangement assay.

### 4.6. Localization Analysis of AtPRK6-mRuby and AlPRK6-mRuby

Pollen tubes were grown through cut styles on pollen tube growth medium for 4.5 h at 22 °C in the dark. Confocal images were acquired using an inverted microscope (IX81; Olympus) equipped with a spinning disk confocal scanner (CSU-X1; Yokogawa Electric Corporation, Japan), 561-nm LD lasers (Sapphire; Coherent, Santa Clara, CA, USA), and an EM-CCD camera (Evolve 512; Photometrics, Tucson, AZ, USA). A 40× water immersion objective lens (UApo/340; Olympus, Japan) and 1.6× intermediate magnification changer were used.

## Data Availability

The authors confirm that the data supporting the findings of this study are available within the article and its Supplementary Materials.

## Supplementary Materials

The following supporting information can be downloaded at: https://www.mdpi.com/article/10.3390/ijms23052722/s1.
