# Supplementary material for: Quantification of Species-Preferential Micropylar Chemoattraction in Arabidopsis by Fluorescein Diacetate Staining of Pollen Tubes
Source: Int J Mol Sci. 2022 Feb 28;23(5):2722. doi: 10.3390/ijms23052722 (PMC8910611; doi:10.3390/ijms23052722)
Supplement: Supplementary file 1 [file ijms-23-02722-s001.zip › ijms-1583415-supplementary.pdf]

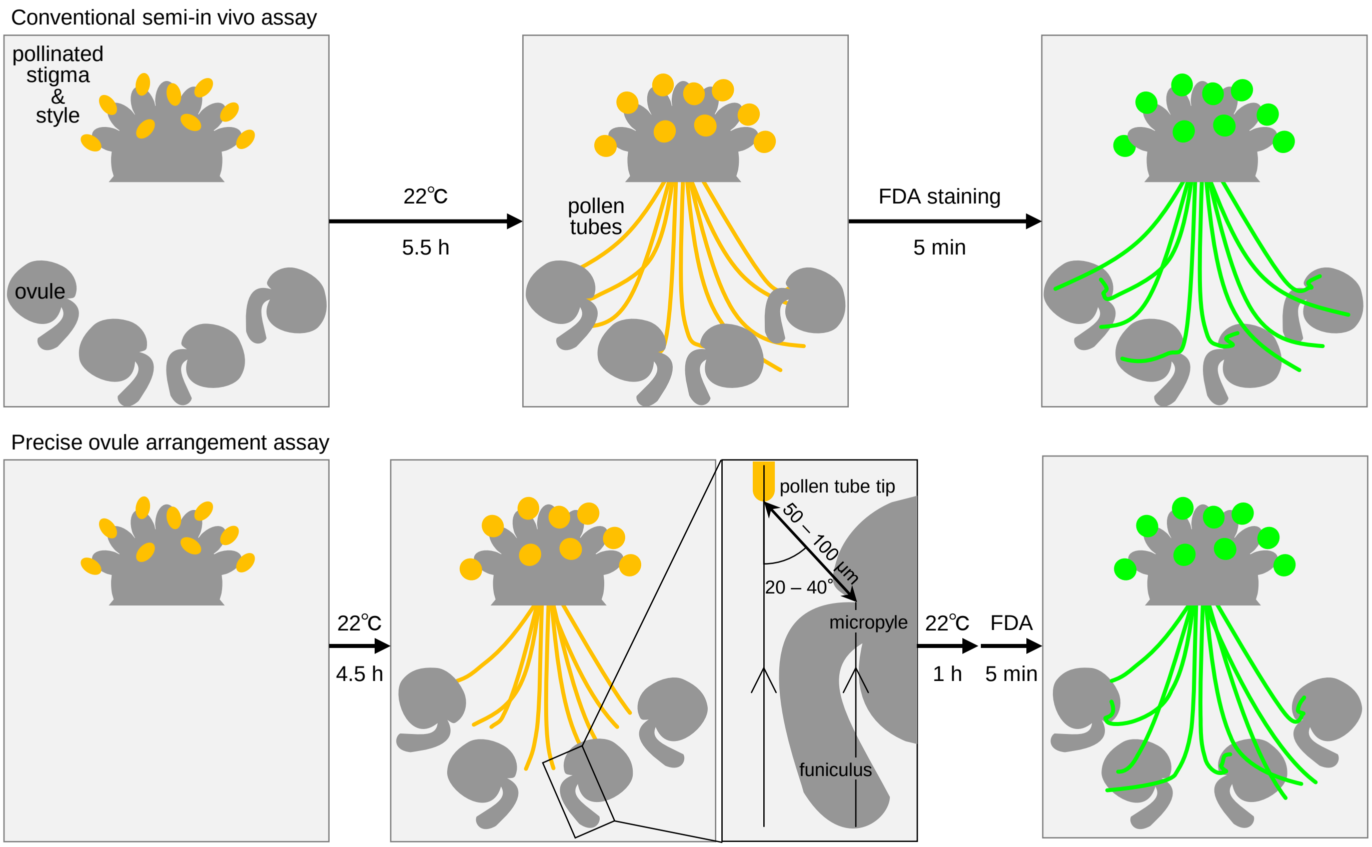

Supplementary Figure S1. Schematic diagram of conventional semi-in vivo assay and precise ovule arrangement assay.

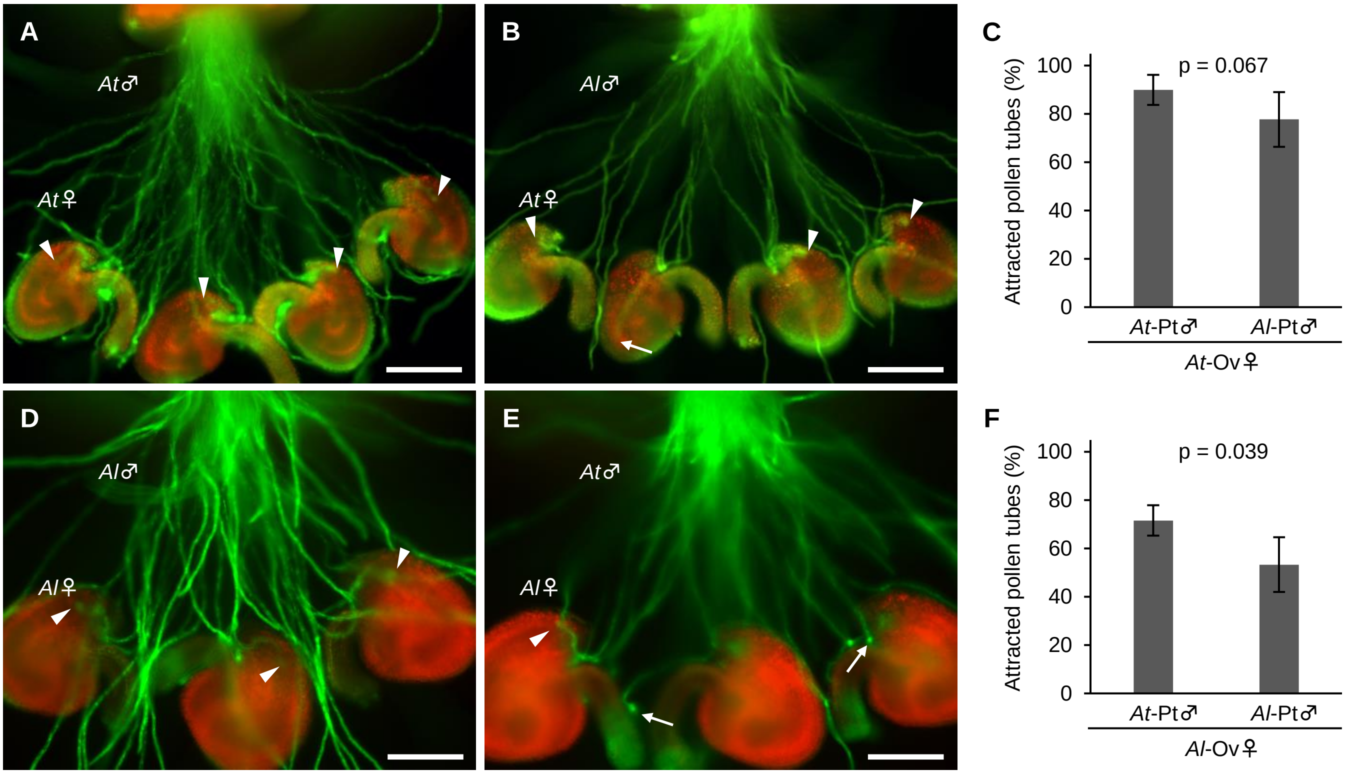

Supplementary Figure S2. Conventional semi-*in vivo* assay for *Arabidopsis* species.

A–C Conventional semi-*in vivo* assay with *A. thaliana* (*At*) and *A. lyrata* (*Al*) pollen tubes for *A. thaliana* ovules. Rates of attracted pollen tubes (Pt) of *A. thaliana* (**A**) and *A. lyrata* (**B**) by *A. thaliana* ovules (Ov) are shown in (**C**). **D–F** Conventional semi-*in vivo* assay with *A. thaliana* and *A. lyrata* pollen tubes for *A. thaliana* ovules. Rates of attracted pollen tubes of *A. thaliana* (**D**) and *A. lyrata* (**E**) by *A. lyrata* ovules are shown in (**F**). Arrowheads indicate the tips of attracted pollen tubes. Arrows indicate the tips of unattracted pollen tubes. Each assay was replicated five times with 11–16 ovules. P-values were calculated by Fisher's exact test. Data are mean ± SD. Scale bars, 200 μm.

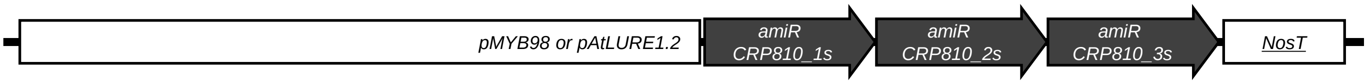

amiRNA-CRP810_1s	3’	CCCAUUCAUAUAAGCAUGUAU	5’
CRP810_1.1 (302-322)	5’	AGATAGGTATATTCGCACATG	3’
CRP810_1.2 (299-319)	5’	GGGTAAGTATATTCGTACATG	3’
CRP810_1.3 (302-322)	5’	GGGTAAGTATATTCGTACATG	3’
CRP810_1.4 (299-319)	5’	GGGTAAGTATATTCGTACATG	3’
CRP810_1.5 (299-319)	5’	GGGTAAGTATATTCGTACATG	3’
CRP810_1.6 (301-321)	5’	GGGTAAGTATATTCGTACATG	3’
amiRNA-CRP810 2s	3’	UUCGCUUGCUUGUAUAACCUU	5’
CRP810_2.1 (200-220)	5’	CAGCATACGAACATATTGGAC	3’
CRP810_2.2 (238-258)	5’	CAGCACACGAACATATTGGAC	3’
CRP810_2.3 (219-239)	5’	CAGCGCACGAACATATTGGAC	3’
amiRNA-CRP810 3s	3’	AUCCGCAUUACUCAAAAACAU	5’
CRP810 3.1 (278-298)	5’	AAGGCGTAATGAGTTTTTGTT	3’
CRP810_3.2 (291-311)	5’	AAGGCGTAATGAGTTTTTGTT	3’

Supplementary Figure S3. Vector construction, targeted DNA sequences, and artificial microRNA (amiRNA) precursor sequences to knock down genes *CRP810_1s*, *CRP810_2s*, and *CRP810_3s*.

Primers Name	Sequences(5'-3')
prk6-1 Left primer	ATCCTGACTTCCACAACATCG
prk6-1 Right primer	ACCCTTTCCTTCTCAACCTTC
myb98-1 Left primer	TGTTTACCCCAAATTCAACAACACC
myb98-1 Right primer	TCCACTGCTTGGAAATGATGATG
crp810_4-1 Left primer	TAGGTACCTTACAATCCCACACCAATAG
crp810_4-1 Right primer	TAAGGATCCATGAGGACAATTGTTTTGTTTTC
ms1 Left primer	ACCGGTTTAAAGGCCAGGTAC
ms1 Right primer	CTGCCTCTCCGTACACTTCAC
LBb1.3 (Light Border primer)	ATTTTGCCGATTTCGGAAC

Supplementary Table S1. Primers used in this study for genotyping.
